# Supplementary material for: Assessment of neonatal thermal cares: Practices and beliefs among rural women in West Guji Zone, South Ethiopia: A cross-sectional study
Source: PLOS Glob Public Health. 2022 Jun 15;2(6):e0000568. doi: 10.1371/journal.pgph.0000568 (PMC10021890; doi:10.1371/journal.pgph.0000568)
Supplement: S1 File — (DOCX) [file pgph.0000568.s001.docx]

**S1 File: English version questionnaire (Quantitative tool)**

**Note:** This questionnaire has been developed for the study after reviewing relevant literatures. It is original work developed by the authors of the manuscript. Authors authorize unrestricted use by any interest party provided that the source is due acknowledged.

**Code of questionnaire: _________________**

**Part I: Socioeconomic and demographic characteristic of respondents**

| **No.** | **Question** | **Answer** | **Skip to** |
| --- | --- | --- | --- |
| 1 | Age of mother | __________(age in completed years) |  |
| 2 | Age of infant | __________(age in month) |  |
| 3 | Sex of infant | 1. Male 2. Female |  |
| 4 | Religion of mothers | 1. Waaqefata 2. Protestant Christian 3. Orthodox Christian 4. Islam 5. Catholic Christian 6. Others ________________(specify ) |  |
| 5 | Marital status of mother | 1. Single 2. Married 3. Separated 4. Divorced 5. Widowed |  |
| 6 | Occupation of mother | 1. Farmer 2. Housewife 3. Merchant 4. Daily laborer 5. employed 6. Others_________________(specify) |  |
| 7 | Education status of mothers | 1. Cannot read and write 2. read and write only, but no formal schooling 3. Primary 1^st^ cycle(1-4 grade) 4. Primary 2^nd^ cycle(5-8 grade) 5. Secondary school (9-12 grade) 6. College and above |  |

**Part II: Past obstetric and reproductive history and Health care utilization**

| **No.** | **Question** | **Answer** | **Skip** |
| --- | --- | --- | --- |
| 1 | Parity of mother | __________(in number ) |  |
| 2 | Place of delivery(the youngest infant ) | 1. Home 2. Health post 3. Health center 4. Hospital 5. Other_______________(specify) |  |
| 3 | If the answer to Q. 2 is home, How many attendants were present during delivery? | _________(number of attendants ) |  |
| 4 | Who attended the delivery(the youngest infant) | 1. TBA 2. Relatives 3. HEWs 4. Health professionals 5. None 6. Others(specify)______________ |  |
| 5 | Did you visit ANC while pregnant for your youngest baby? | 1. Yes 2. No |  |
| 6 | If the answer to Q.5 is yes, from where did you get ANC? | 1. Health post 2. Health center 3. Hospital 4. Other_______________(specify) |  |
| 7 | What was gestational age of the fetus when you gave birth to your youngest baby? | 1. _______(gestational age in month) 2. Unknown 3. Others(specify)__________ |  |
| 11 | What was weight of your youngest baby when you gave birth | 1. _____(birth weight in kg) 2. Unknown 3. Others(specify)___________ |  |

**Part III: Neonatal thermal care practices characteristic of respondents**

| 1 | When did you dry off birth fluids from your newborn after delivery(the youngest baby) | 1. Immediately before placenta delivery 2. Immediately after placenta delivery 3. Delayed after placenta delivery 4. Do not know |  |
| --- | --- | --- | --- |
| 2 | After how many minutes did you dry off birth fluids from your newborn after delivery(the youngest baby) | 1. Less than five minutes/immediately. 2. 5 to 15 minutes 3. 16 to 30 minutes 4. More than 30 minutes 5. Do not know |  |
| 3 | If your answer to Q.2 is 1 or 2 or 3, why drying the newborn was delayed? | 1. Birth attendant/s focusing on the mother until the placenta was delivered 2. Waiting for the cord to be cut 3. Waiting for placenta to deliver 4. Taboo for caring for newborn until placenta is buried 5. No one is present 6. Waiting for the baby to be bathed. |  |
| 4 | Did you prepare towel or cloth for drying newborns when delivery date approached? | 1. Yes 2. No |  |
| 5 | Do you believe immediate drying helps in keeping the newborn warm? | 1. Yes 2. No 3. Do not know |  |
| 6 | After how many minutes did you wrap your newborn after delivery(the youngest baby) | 1. Less than five minutes/immediately 2. 5 to 15 minutes 3. 16 to 30 minutes 4. More than 30 minutes 5. Do not know |  |
| 7 | If your answer to Q.6 is 1 or 2 or 3, why wrapping the newborn was delayed? | 1. Birth attendant/s focusing on the mother until the placenta was delivered 2. Waiting for the cord to be cut 3. Waiting for placenta to deliver 4. Taboo for caring for newborn until placenta is buried 5. No one is present 6. Waiting for the baby to be bathed. |  |
| 8 | Did you prepare warm clothes for wrapping newborns when delivery date approached? | 1. Yes 2. No |  |
| 9 | Do you believe immediate wrapping helps in keeping the newborn warm? | 1. Yes 2. No |  |
| 10 | When did you give first bath to your newborn after delivery? (the youngest baby) | 1. __________hrs. after delivery 2. Other___________(specify ) |  |
| 11 | If the answer to question **10** is less than 6 hour, Why did you bath the newborn earlier? | 1. To remove different secretion from body like dirty fluid or blood 2. to remove visible vernix 3. to improve health and strength of newborn 4. making baby refreshed and comfortable 5. Encouraging sleep and improving health 6. To prevent different smells 7. Other____________________(specify) |  |
| 12 | If the answer to question 10 is 6 hours or more, Why did you delay bathing the newborn? | 1. Need to keep the baby warm 2. Health worker advised me to do 3. Other____________________(specify) |  |
| 13 | Do you believe delaying first newborn bath by at least 6 hours helps in keeping newborn warm? | 1. Yes 2. No 3. Do not know |  |
| 14 | What type of water did you use to give first bath to your youngest baby? | 1. Warm water 2. Cold water 3. Other____________________(specify) |  |
| 15 | Did you put your youngest baby in skin to skin contact to you immediately after delivery or in the first week of delivery to keep him/her warm?(**i.e** **Show picture**) | 1. Yes 2. No |  |
| 16 | If your answer to Q 15 is yes , on which day after delivery you had skin-to skin contact with your baby? | 1. _____________(write exact day) 2. Other____________________(specify) |  |
| 17 | If your answer to Q. 15 is no , why? ( MORE THAN ONE ANSWER IS POSSIBLE) | 1. It facilitates disease transmission 2. May hurts baby’s cord 3. Baby is delicate-chest/bone/heart gets hurt 4. It disrupts mothers to rest/ causes exhaustion 5. Due to activities taking place after birth/no time for skin to skin contact 6. I do not know such practice 7. Other______________(specify) |  |
| 18 | If your answer for Q15 is yes, Why you put baby in skin to skin contact? | 1. Having immediate access and feeling close to the baby 2. Starting breastfeeding quickly 3. I think it teaches us to start loving our baby from the very beginning 4. Health workers advised me to do 5. To keep baby warm 6. Other_______________(specify) |  |
| 19 | Do you believe keeping newborn in skin to skin contact with mother during first week of delivery helps in keeping newborn warm? | 1. Yes 2. No 3. Do not know |  |
| 20 | Did you put baby hat/head cover on head of your youngest baby immediately after delivery? | 1. Yes 2. no |  |
| 21 | If answer to Q.20 is yes, why you put head cover/hat on your newborn? | ____________________________________(write answer) |  |
| 22 | If answer to Q.20 is no, why you did not put baby hat/head cover on head of your youngest baby? | ____________________________________(write answer) |  |
| 23 | Do you usually used to put baby hat/head cover on your youngest baby during first month of delivery? | 1. Yes 2. No |  |
| 24 | Did you prepare baby hat/head cover to your youngest baby, when delivery date approached or when labor started? | 1. Yes 2. No |  |
| 25 | Do you believe that always putting baby hat/head cover on newborn head during first month of delivery helps in keeping newborn warm? | 1. Yes 2. No 3. Do not know |  |
| 26 | Did you breastfeed your youngest baby after delivery? | 1. Yes 2. No |  |
| 27 | How much hours after delivery, did you first put your baby to the breast? | ____________(Hrs.) |  |
| 28 | What did you do with first breast milk secreted from your breast called colostrum? | 1. Provided to newborn 2. Milk out and/discard 3. Other ____________________(specify) |  |
| 29 | If answer to Q.27 is greater than 1 hr., why did you delay breast feeding initiation? | 1. Colostrum is dirty/not good for baby 2. Lack of sufficient breast milk 3. Baby need sleep/rest after delivery 4. Baby did not show sign of hunger 5. Baby needed to be bathed 6. Mother needed rest 7. Mother needed bathing 8. Other___________________(specify) |  |
| 30 | Did you provide any fluid/food (*except medicine*) to your newborn during first month of delivery? | 1. Yes 2. No 3. Do not know |  |
| 31 | Do you believe early initiation of breastfeeding within 1 hour of delivery helps in keeping newborn warm? | 1. Yes 2. No 3. Do not know |  |
| 32 | Do you believe frequently breastfeeding newborn during first month of delivery helps in keeping newborn warm? | 1. Yes 2. No 3. Do not know |  |
