# Supplementary material for: Assessment of neonatal thermal cares: Practices and beliefs among rural women in West Guji Zone, South Ethiopia: A cross-sectional study
Source: PLOS Glob Public Health. 2022 Jun 15;2(6):e0000568. doi: 10.1371/journal.pgph.0000568 (PMC10021890; doi:10.1371/journal.pgph.0000568)
Supplement: S2 File — (DOCX) [file pgph.0000568.s002.docx]

**S2 File: Focus Group Discussion guide (for Mothers of Infants less than 6 months old)**

**Note:** This FGD guide has been developed for the study after reviewing relevant literatures. It is original work developed by the authors of the manuscript. Authors authorized unrestricted use by any interest party provided that the source is due acknowledged.

**Guide Questions**

1. Do you think newborns should be kept warm after delivery? Why?
2. What do you think are benefits of keeping newborns warm?
3. How newborn are kept warm after delivery in your area?
4. How commonly women in your area wrap and dry their newborns immediately after delivery? What do you believe at it?
5. How commonly women in your area cover head of newborns immediately after delivery and within first month of delivery? Probe head covering within first month after delivery? What do you believe about head covering?
6. How commonly women in your area practice skin-to-skin care for newborns immediately after delivery? Probe: practices within first week of delivery[**please show picture**]. What do you believe about skin to skin care of newborn?
7. When commonly newborns are bathed after delivery in your area? (Probe: immediately, after X hours of delivery)? Why? Probe: in relation to placenta delivery time.
8. When commonly newborns are breastfed after delivery in your area? (Probe: immediately, after X hours of delivery)? Why? Do you believe that early breast feeding helps in keeping baby warm?
