# Supplementary material for: Assessment of neonatal thermal cares: Practices and beliefs among rural women in West Guji Zone, South Ethiopia: A cross-sectional study
Source: PLOS Glob Public Health. 2022 Jun 15;2(6):e0000568. doi: 10.1371/journal.pgph.0000568 (PMC10021890; doi:10.1371/journal.pgph.0000568)
Supplement: S3 File — (DOCX) [file pgph.0000568.s003.docx]

**S3 File: Afaan Oromo version questionnaire (Quantitative tool)**

**Note:** This questionnaire has been developed for the study after reviewing relevant literatures. It is original work developed by the authors of the manuscript. Authors authorize unrestricted use by any interest party provided that the source is due acknowledged.

**Lakkoofsa/koodii Hirmaata: _________________**

**Kuta I: Odeeffanno haala hawaas-dinaagde**

| **lak.** | **Gaafi** | **Deebi** |
| --- | --- | --- |
| 1 | Umri haadha | __________(umrii wagga guutuudhaan) |
| 2 | Umrii daa’ima | __________(umrii ji’aan) |
| 3 | Saala daa’ima | 1. Dhiira 1. Dubra |
| 4 | Amaantii Haadha | 1. Waaqefata 2. Kiristaana Pirotestaantii 3. Kiristaana Ortodoksii 4. Islaama 5. Kiristaana Katolikii 6. Ka bira ________________(barressi) |
| 5 | Haala gaa’ila haadha | 1. Kan hin heerumini 2. Gaa’ila qabdii 3. Abbaa warra wajjin hin jirre, garuu wal hin hiiknee 4. Heerumte kan hiiktee 5. Abbaan warra kan irra du’ee |
| 6 | Hojii haadha kan ijoo | 1. Qonnaan bultu 2. Haadha Mana 3. Daldaaltu 4. Hojii guyya guyya 5. Qacaramtuu 6. Ka bira________________(barressi) |
| 7 | Sadarka barumsa haadha | 1. Dubbisu fi barressu kan hin dandeenne 2. Dubbissu fi barressu danadetti, garuu hin baranne. 3. Sadarka 1ffa (kuta 1-4) 4. Sadarkaa 1ffa ( kuta 5-8) 5. Sadarkaa 2ffa (kuta 9-12) 6. Sadarka kollejji fi isa oli |

**Kuta II: Haallan ulfa, fayya wal hormaata fi fayyadaminsa tajaajila fayyaa.**

| **lak.** | **Gaafi** | **Deebi** |
| --- | --- | --- |
| 1 | Hanga hardhaat yeroo meeqa deesse? | __________(lakkoofsadhaan ) |
| 2 | Bakka dahumsa (**daa’ima dhuma** ) | 1. Mana 2. Keella fayya 3. Buufata fayyaa 4. Hoospitaala 5. Ka bira_______________(barressi) |
| 3 | Yoo deebiin **G.2** mana tahee, nama meeqatu dahumsa irrat si gargaare? | _________(lakkofsa nama) |
| 4 | Enyuutu sii deesise (**daa’ima dhuma**) | 1. Deessiftu Aadaa 2. Fira 3. Eksiteenshini fayyaa 4. Ogeessa Fayyaa 5. Namu hin ture 6. Ka bira______________(barressi) |
| 5 | Yeroo daa’ima maandha ulfaa qabduu, ulfaa keetif hordoffi dahumsa dura goote? | 1. Eyye 2. Miti |
| 6 | Yoo deebiin **G.5** eyye tahee, hordoffi essat goote? | 1. Keella fayyaa 2. Buufata fayyaa 3. Hoospitaala 4. Ka bira_______________(barressi) |
| 7 | Yeroo daa’ima maandha deessu, ulfi ji’a ,meeqa ture? | 1. _______(ji’a ulficha) 2. Hin beekkamu 3. Ka bira_______________(barressi) |
| 11 | Daa’immi maandha yeroo dhalatu ulfatinni isa/ishi meeqa? | 1. _____(ulfatina Kg dhaan) 2. Hin beekkamu 3. Ka bira____________(barressi) |

**Kuta III: Haallan Haadholiin daa’imma didiqqo hoo’ina qaama ittiin eegan.**

| **Lak** | **Gaafi** | **Deebi** |
| --- | --- | --- |
| 1 | Yeroo daa’ima maandha deessu, qaama daa’ima huccuudhaan jalqabaaf yeroo kam goggosite? | 1. Battalumati osoo obbatiin hin bahin 2. Battalumati erga obbatiin baheen booda 3. Ture erga obbatiin baheen booda 4. Hin yaadadhu |
| 2 | Yeroo daa’ima maandha deessu, qaama daa’ima huccuudhaan yeroo jalqabaaf daqiiqa meeqa booda goggosite? | 1. Daqiiqa 5 gadit/battalumati. 2. Daqiiqa 5 -15 keessat 3. Daqiiqa 16-30 keessat 4. Daqiiqa 30 olit 5. Hin yaadadhu |
| 3 | Yoo deebiin **G.2,** 1ykn 2 ykn 3 tahee, daa’ima qaama osoo hin goggosin maaliif turtan? | 1. Dessiftuun haadha irrat xiyyeffacha turtee hanga obbaatiin bahuut. 2. Handhura hangaa kutanit egnee 3. Hanga obbaatiin bahuut egnee 4. Hangaa obbaatiin awwalamut daa’ima goggosuun safuu dha. 5. Namu bakka hin turee waan taheef 6. Hanga da’ima qaama dhiqaan egnee. |
| 4 | Yeroo daa’ima maandha dahuuf dhiyaatuu huccuu daa’ima ittin gogsitu kopheffate turte? | 1. Eyye 2. Miti |
| 5 | Daa’imni akkuma dhalateen battalumat goggosuun hoo’ina qama daa’ima eeguuf ni gargaara jette amantaa? | 1. Eyye 2. Miti 3. Hin beeku |
| 6 | Yeroo daa’ima maandha dessuu, yeroo jalqabaaf daqiiqa meeqan booda huccu qorra ittisuun aguugdee? | 1. Daqiiqa 5 gadit/battalumati. 2. Daqiiqa 5 -15 keessat 3. Daqiiqa 16-30 keessat 4. Daqiiqa 30 olit 5. Hin yaadadhu |
| 7 | Yoo deebiin **G.6,** 1 ykn 2 ykn 3 tahee, daa’ima huccu qorra ittisuun osoo hin aguugin maaliif turtan? | 1. Dessiftuun haadha irrat xiyyeffacha turtee hanga obbaatiin bahuut. 2. Handhura hangaa kutanit egnee 3. Hanga obbaatiin bahut egnee 4. Hangaa obbaatiin awalamuut daa’imma aguuguun safuu dha. 5. Namu bakka hin turee waan taheef 6. Hanga daa’ima qaama dhiqan egnee. |
| 8 | Yeroo daa’ima maandha dahuuf dhiyaatuu huccuu qorra kan daa’ima ittin aguugdu kopheffate turte? | 1. Eyye 2. Miti |
| 9 | Daa’imni akkuma dhalateen battalumat huccun aguuguun hoo’ina qama daa’ima eguuf ni gargaara jette amantaa? | 1. Eyye 2. Miti 3. Hin beekuu |
| 10 | Erga daa’ima deesse booda sahati meeqaa turtee yeroo jalqabaaf qaama daa’ima dhiqxee?(**daa’ima maandha**) | 1. Sahati__________booda 2. Ka bira______________(barressi ) |
| 11 | Yoo deebiin **G.10**, Sahat 6 gad tahee, maaliif daa’ima ariifaattani dhiqxan?  (**deeebii tokko ol filuun ni danda’ama**) | 1. Dhangala’a xuraawa kan akka dhiiga qaama irra qulqulleessuuf 2. Qoofira ykn wanta adii qaama irra qulqulleessuuf 3. Fayya fi jajjabina daa’imaaf 4. Daa’imni akka itti mijaa’uuf 5. Hirriba daa’ima foyyessuf 6. Fooliiwwan adda adda balleessuuf 7. Ka bira___________________(barressi) |
| 12 | Yoo deebiin **G.10**, Sahat 6 fi isa oli tahee, maaliif daa’ima akkas tursitani dhiqxan? | 1. Daa’imni hoo’inaan akka turuf 2. Oggessa fayyaat akka tursinu nu gorse 3. Ka bira___________________(barressi) |
| 13 | Daa’imni erga dhalateen booda yoo diqqaatee sahati 6 tursani dhiquun hoo’ina qaama daa’ima eeguf ni gargaara jette amantaa? | 1. Eyye 2. Miti 3. Hin beeku |
| 14 | Daa’ima maandha erga dhalateen booda bishaan akkamitiin yeroo jalqabaaf dhiqxe? | 1. Bishaan Hoo’aa/Bulluqaa 2. Bishaaan qabbana’a 3. Ka bira__________________(barressi) |
| 15 | Daa’ima maandha akkuma dhalateen ykn bulii torba kessati hoo’ina isaa eguuf qaama isa/ishee qaama kee wajjin waliit qabdee turtee? (**Fakki argisisi**) | 1. Eyye 2. Miti |
| 16 | Deebiin **G.15**, eyye yoo tahee, guyya meeqaffa irrati qaama da’ima fi qaama kankee waliti qabdee turtee? (**Yeroo jalqabaatiif** ) | 1. _____________(guyyicha barressi ) 2. Ka bira___________________(barressi) |
| 17 | Deebiin G.15, miti yoo tahee, maaliif?( **Deebii tokko ol filuun ni dandahama**) | 1. Dhukkubni akka dadarbuuf haala mijeesa. 2. Handhuraa daa’ima midhuu danda’a 3. Laphe/laffe daa’ima miidhu danda’aa 4. Haadha boqonna dhowwa/dadhabsiisa 5. Hojii biran qabamnee/ yeroon hin turre 6. Qaama kiyya fi kan daa’iama walit qabu hin beeku ture 7. Ka bira______________(barressi) |
| 18 | Deebiin G.15, eyye yoo tahee, maaliif qaama daa’imaa fi kankee walit qabdee? ( **Deebii tokko ol filuun ni dandahama**) | 1. Daa’imaan waliit dhiyaachuuf ykn qaama kiyyat akka dhiyatuu gochuuf. 2. Dafnee harma hoosisuu jalqabsisuuf. 3. Jaalala daa’imaaf akkaan qabadhuu naa godha jedheen amana 4. Oggessa fayyat akkasit na gorsee 5. Hoo’ina qaama daa’ima eeguuf. 6. Ka bira__________________(barressi) |
| 19 | Daa’imni erga dhalateen booda hanga bulii torba qaama hadhaat maxxansani qabuun hoo’ina qaama daa’ima eeguuf ni gargaara jette amantaa? | 1. Eyye 2. Miti 3. Hin beeku |
| 20 | Daa’ima kan kee maandha akkuma dhalateen koofiya/gonfoo/qoobii mata irra ketteefi turtee? | 1. Eyyee 2. Miti |
| 21 | Deebiin **G.20**, yoo eyye tahee, maaliif koofiya/gonfoo/qoobii daa’imichaf goote? | ____________________________________(Deebi barressi) |
| 22 | Deebiin **G.20**, yoo miti tahee, maaliif koofiya/gonfoo/qoobii daa’imichaf hin godhin? | ____________________________________(Deebi barressi) |
| 23 | Yeroo bay’ee daa’imma kankee maandhaatiif hanga ji’a tokko gutuut koofiya/gonfoo/qoobii ni goota turtee? | 1. Eyye 2. Miti |
| 24 | Yeroo daa’ima maandha dahuuf dhiyaatuu koofiya/gonfoo/qoobii daa’imaaf kophessite turte? | 1. Eyye 2. Miti |
| 25 | Daa’imni erga dhalateen booda hanga bulii ji’aa tokko yeroo mara koofiya/gonfoo/qoobii mata irra godhuun hoo’ina qama daa’ima eguuf ni gargaara jette amantaa? | 1. Eyye 2. Miti 3. Hin beeku |
| 26 | Daa’ima kankee maandha erga dhalateen bood harma hoosiiftee? | 1. Eyye 2. Miti |
| 27 | Daa’ima kankee maandha erga dhalateen booda sahati meeqaan booda harma jalqaba kenniteef? | ____________ (Sahati.) |
| 28 | Deebiin **G. 27** sahati 1 ol yoo tahee,harma hosisuu jalqabsisuuf maaliif akkas turtaan? | 1. Anani jalqaba xurawa/daa’imaaf dansa miti. 2. Harmi koo annan gaha hin qabu ture 3. Daa’imni rafaa/boqocha waan tureef 4. Daa’imni mallatto bela’uu wan hin argisifneef 5. Daa’imni dhiqamuu waan qabuuf 6. Haadhi boqonna waan barbaduuf 7. Haadhi qama dhiqachuu waan barbaduuf 8. Ka bira___________________(Barressi) |
| 29 | Anaan harma kan jalqaba bahuu, jajjabaa sun maal goote? (**yeroo daa’ima maandha dessu**) | 1. Daa’imaaf keenne 2. Alat elme, jiigsee 3. Ka bira __________________(barressi) |
| 30 | Daa’ima kankee isaa maandhaf hanga ji’a tokko guutut annani harmatiin alaa dhangala’a ykn nyaata biro kenniteefi beekta?(**Qoricha mana yaala hin dabalatu**) | 1. Eyye 2. Miti 3. Hin yaadadhu |
| 31 | Daa’imni erga dhalateen booda sahati tokko osoo hingunne harma hosisuun hoo’ina qaama daa’ima eguuf ni gargaara jette amantaa? | 1. Eyyen 2. Miti 3. Hin beeku |
| 32 | Daa’imni erga dhalateen hanga ji’aa tokko guutut guyyaati yeroo baay’ee harma hosisuun hoo’ina qama daa’ima eguuf ni gargaara jette amantaa? | 1. Eyye 2. Miti 3. Hin beeku |
| 33 | Daa’imni hanga ji’a tokko guutut annani harma qofa hoosisuun osoo dhangala’a ykn nyaata biro hin daballe hoo’ina qaama daa’ima eguuf ni gargaara jette amantaa? | 1. Eyye 2. Miti 3. Hin beeku |

**Baay’ee Galatooma!!!**
