# Supplementary material for: Assessment of neonatal thermal cares: Practices and beliefs among rural women in West Guji Zone, South Ethiopia: A cross-sectional study
Source: PLOS Glob Public Health. 2022 Jun 15;2(6):e0000568. doi: 10.1371/journal.pgph.0000568 (PMC10021890; doi:10.1371/journal.pgph.0000568)
Supplement: Attachment — Submitted filename: Response to Reviewers.docx [file pgph.0000568.s004.docx]

**Response to editor and Reviewers**

**Editor’s Comments and authors’ responses**
1. Please see the PLOS Data Policy and consider whether the data underlying this work can be made publicly available. See specifically the FAQ on why single authors are not recommended as the point of contact for work submitted.

Response: All relevant data are included into the manuscript including tables.

2. Please consider adding to the conclusions section of your abstract the possible public health implications of your research findings.

Response: we have added possible public health implication of the research findings into the abstract of the manuscript which read as “Interventions to familiarize skin to skin care and reduce misconceptions surrounding it should be in place to improve thermal care of high risk newborns. “

3. Please provide detail about the women who were not successfully interviewed - did they decline participation or were they not contacted? If they declined, were there any key differences between the sample who chose to participate and those who declined?

Response: Thirty five women were not available within their home for the interview after two home visits.

**Reviewers’ Comments and authors’ responses**

1. Reviewer #1: This paper is interesting to read and publishable. It has some grammatical errors that can be worked on. I have also added some comments that should be reviewed and updated before publishing. This will be new information for scientists to learn from.

Response: we have proofread the document again and corrected all grammatical errors that we found. We have also incorporated all suggested revisions.

2. Reviewer #2: Thanks for the opportunity to revise this paper and congratulations to the authors for submitting it. This paper has interesting findings on newborn thermal care practices that could be potentially used to improve newborn care and prevent newborn mortality. It also sheds light on the local practices of a population barely present in the international literature. However, some further work is needed to make it suitable for publication. Here are my recommendations
General: The main issue I see is that the paper was supposed to “assessed practices about neonatal thermal cares (early drying and wrapping, head covering, skin to skin care, newborn bath time and early breastfeeding initiation) and related beliefs among rural women in West Guji Zone, South Ethiopia”. I do not think that the “related beliefs” have been deeply explained except for a few quotes referring to cold prevention. An exception to this is the paragraph on lack of skin-to-skin care. There is no clear understanding of why participants think that wrapping and drying the baby is important or why it has to be done after the first bath. These types of reasons are mentioned in the survey results (Tables 3, 4, and 5) but the main reason to have a qualitative arm of a project like this is to explore these reasons and the customs/beliefs attached to it.

Response: We have re-presented the results in as detail as possible with regard to beliefs and reasons surrounding thermal care practices by including results from both survey and FGDs.

3. I also recommend that the paper is proofread by a native English speaker. Abstract: I think you meant “quantitative” when you wrote: “The qualitative data were cleaned, coded, and analyzed by SPSS version 20”.

Response: we have proofread the document again and corrected all grammatical errors that we found. Typo problem in abstract which stated “Qualitative “is corrected to “quantitative”

4. Introduction:
In the end, the authors say “Few studies have assessed neonatal thermal care practices in rural Ethiopia”. Could you cite some of these? How is your study different than those?

Response: There is no previous study which assessed newborn thermal care practices in rural Ethiopia. Therefore, we have revised our statement and now it read like “No previous study has assessed neonatal thermal care practices in rural Ethiopia”

5. Either here or in the discussion, could you mention if there are institutional (governmental or non-governmental) programs or policies aimed at improving newborn thermal care?

Response: Yes, we have included government program which aims to improve thermal protection of newborns in Ethiopia under introduction section.

6. Explain the “Harmful cultural norms and traditional practices that affect neonatal thermal care practices at home exist in different contexts of Africa [14]”. What are the known challenges in Ethiopia or the region regarding neonatal thermal care?

Response: We have added regional challenges to neonatal thermal care.

7. Methods:
Sampling procedures: what are the different climate areas? Why are they important to determine a sampling process? Do they correspond to different ethnicities? Different language? Since we are talking about thermal care practices, can the differences in climate between these areas affect how they take care of their newborns’ temperature after being born?

Response: Three climatic areas are called Dega, Woinedega, and Kolla. We have included them into sampling procedures section. We undertake sampling procedures in way which ensures fair representation of women from all three climate areas as we were not sure if climate affects practice of thermal care of newborns.

8. Data collection instruments and procedures: how were the FGDs participants recruited? Did any of the participants receive compensation for their time? Were the FGDs conducted in Afaan Oromo? Is that the native language of all the FGDs participants? Is the principal investigator fluent in that language? To what language were the transcripts translated? How long were the FGDs? Where did they take place?

Response: FGD participants were recruited purposively to ensure variation in age, education and Socio-economic status. This has been mentioned in sampling procedures section of the manuscript. FGD participants did not receive compensation for their time. FGDs were conducted in Afaan Oromo, which is native language of all FGD participants. All research teams who participated in FGD facilitation are fluent in Afaan Oromo. Transcripts were translated into English. All FGDs took between 90 to 120 minutes. FGDs were conducted in community settings where only FGD participants, facilitator and moderate presented. All these have been incorporated into the manuscript.

9. Quality assurance: “All errors in transcription and translation those were found were corrected.” What type of errors are you referring to? In what context were transcripts revised? During coding?

Response: we are referring to errors like spelling errors, punctuation errors, and errors in stating actual message delivered by FGD participants. We revised transcripts if the content is different from audios and/or field notes. We did not encounter error during coding.

10. Results:
-Based on the results in Tables 3, 4, and 5, and the TGDs guide (File 2), the authors seem to have some interesting data on why women have the newborn thermal care practices that they have. Even if that is part of the goals of the paper, it is not analyzed carefully. Authors have done a great job describing when newborn thermal care practices happen but little is done to explain the reasons either practical (e.g., the baby has to wait to be dried because there is no one to do it while the placenta is being delivered) or cultural behind it. I think the authors should have a section on that where they combine both the qualitative and quantitative data about why people wait until the first bath to apply thermal care practices.

Response: We have analyzed time of wrapping and drying in relation to first bath, and we have presented both qualitative and quantitative findings together with regard to why bath is done before drying and wrapping. We have also presented qualitative and quantitative findings together with regard reasons for early bath, and reasons for head covering.

11. I recommend more consistency in how results are presented. For example, in the first paragraph of Women’s Practice of Neonatal Thermal Care, they discuss immediate drying and include both quantitative and qualitative data. However, in the following paragraphs when they discuss other neonatal thermal care practices, they don’t include any qualitative data.

Response: We have presented our results in more consistent manner and in all the rest of paragraphs relevant quantitative and qualitative findings were presented together.

12. Based on my past two comments, I recommend that the results section is restructured. A first subsection could include what participants report doing regarding neonatal thermal care practices in both the survey and the TGDs. This would look like their first paragraph of the Women’s Practice of Neonatal Thermal Care, meaning a combination of rates and quotes about their drying, wrapping, and bathing practices as well as when/if immediate breastfeeding and skin-to skin care take place. In
a separate subsection, I recommend they include the reasons why women perform their neonatal thermal care practices the way they do. To explain this I suggest authors use both the reasons that came up in the survey and those from the TGDs.

Response: We have presented both qualitative and quantitative findings together with regard to reasons why women perform their neonatal thermal care practices in relevant subsection of the results except for head covering (only qualitative data included, quantitative data are not available), early breastfeeding (only qualitative data included, quantitative data are not available) and skin to skin care (not data about reasons available as practice is non-existent in the area)

13. I also encourage the authors to note the differences between the participants’ responses in the survey and the TGDs. For example, keeping the baby from being cold seems to be the main reason for giving the baby an early bath in the qualitative data, but this does not appear in Table 4 (unless it is included in the “To improve health and strength of newborn” category. Even if that is the case, only 5.5% of participants reported that and it seems much more prevalent in the quotes). This could be further discussed in the Discussion section.

Response: Yes, in qualitative data the main reason for early newborn bath is to protect cold in two ways as stated by participants. First if not removed by washing, birth fluid itself is believed to attract cold. Secondly bathing is done early to prepare newborn for wrapping and drying. So it was considered as an intermediate step in process of cold protection. We look for the difference in participants’ response in survey and FGDs. But we believe frequent quotes mentioned by FGD participants about thermal protection care importance of early bath could equate with the following responses from survey in Table 4(remove different secretions or blood or dirty from body, To remove visible vernix, and improve health and strength of newborn). As survey tends to enforce participants to select the best alternative without opportunities to provide detailed reasons for their behaviors, we did not consider responses from survey and FGDs are different.

14. Are there any more reasons behind covering the newborn’s head than preventing cold?

Response: There were two reasons for head covering. They are protection cold and sunlight. Both are included into results now.

15. Head covering is done after the first bath? Before? During?

Response: We did not ask timing of head covering in relation to first bath. So we did not report temporal relation between head covering and first bath.

16. How common among participants were the quotes you provided in the paper? It would be helpful to know if they are representative of most participants and what unique/negative cases came up.

Response: The quotes that we provided in paper are what participants in general agreed with. Based on overall content of the discussions we believe the quotes represent participants. We did not come across any unique or negative cases.

17. If skin-to-skin contact is not a common practice, how do mothers hold their babies? Did you ask that?

Response: We did not ask that and did not include into the manuscript

18. What happened with the baby between birth and the first bath? Are they kept naked? are they hold by someone or placed somewhere? Is that surface clean? Are they wrapped and then unwrapped for the first bath?

Response: In general our findings indicate that, under normal circumstances, following delivery newborns are taken to bath immediately. We did not explored what happened to newborns in short transition between birth and first bath by FGDs and Survey. That is limitation and we have put this as one of limitations of the study in the last part of discussion section. But when placental delivery is delayed or delivery is at heath facility newborns are wrapped and, dried and wrapped respectively without bathing until placental delivery or until women return home from health facility. These have been incorporated in relevant subsection in the results of manuscript.

19. Why does the first bath take place after the delivery of the placenta?

Response: We did not explore the reason why the first bath takes place after the delivery of the placenta in both FGDs and Survey.

20. Unclear: “Women strongly believe that newborns should be bathed as soon as possible after delivery provided that placental delivery is not delayed. Other recommended newborn thermal care like immediate drying and wrapping are usually done after the first newborn’s bath opposite to the standard recommendation. However, newborn’s bath is rarely done before delivery of placenta”.

Response: We have revised the statements and now they read like “Women strongly believe that newborns should be bathed as soon as possible after placental delivery. Focus discussants quoted “if placenta is safely delivered, the newborns should be bathed immediately and kept away from cold”-25 years old and mother of 5 months old infant… “If placenta is immediately delivered, then the baby will be bathed immediately. Then there will be no problem of cold”- 22 years old and mother of 6 months old infant. It is believed that birth secretion/fluid on newborns’ body is hated because of its foul smell and its attraction of cold. Therefore immediate bathing is done to eliminate foul odor of birth fluid and prevent cold. Other recommended newborn thermal cares like immediate drying and wrapping are usually done after the first newborn’s bath in opposite to the standard recommendation. Immediate bath followed by wrapping with warm and clean clothes is believed to prevent cold from newborns”

21. Were there any differences in the participants’ qualitative and quantitative responses based on age, climate area, economic status, religion, and educational level? Whether if there were differences or not, I think it is worth mentioning in the Results sections and even discussing later.

Response: We included women from diverse backgrounds (age, climate area, economic status, religion, educational level, etc) in survey to ensure that quantitative findings are representative of women in the study area. We did not make comparison of response between different groups based on age, climate area, economic status, religion, educational level, etc. because we do not believe that current sample size would not allow robust comparison to be made as we did not consider such comparison during initial sample size calculation. We looked if there were differences in qualitative responses among participants based on climate, age and educational level. But we did not see any difference in response.

22. Did you ask about where did the water for bathing the baby came from (e.g., boiled water? Potable water? River water?)? I am afraid that could compromise the health of the newborn too.

Response: We did not ask the source of the water for bathing and we did not include that into our report.

23. “However, only 32(8.2%) of women dried-off their babies of birth fluids before
delivery of placenta”. Women did or their birth attendants? Who is in charge of wrapping, drying, and bathing the newborn?

Response: There was mistake in writing the statement. Now it read like this “However, only 32(8.2%) of newborns were dried-off birth fluids before delivery of placenta”. we did not ask who actually did wrapping, drying and bathing in both survey and FGDs. So we did not report in our manuscript.

24. Discussion:
-In the first paragraph of the discussion it is worth mentioning that even though drying and wrapping are newborn thermal care practices, they are conducted after the first bath. In case that order compromises the efficiency of those thermal care practices in preventing hypothermia, it should be noted and supported with scientific literature.

Response: We have added the statement “Even though drying and wrapping are common newborn thermal care practices, they are conducted after the first bath”. We also added the risk of early bath before drying and wrapping into discussion.

25. You mention some of that at the end of the second paragraph: “they believe better thermal protection would be achieved if drying and wrapping are preceded by bath”. However, I suggest you also put it in the first paragraph because you are summarizing your findings there. I also recommend a discussion about the risks of drying and wrapping the baby after an early bath, if any.

Response: We have put the statement which “..they believe better thermal protection would be achieved if drying and wrapping are preceded by bath” in the first paragraph of the discussion. We added the risk of early bath before drying and wrapping into discussion.

26. Regarding paragraph 3, are there any other studies about head covering that you could cite and compare to your results as you did in paragraphs 2 and 4?

Response: No previous study reported on the prevalence of head covering.

27. The paragraph on early bathing (#5) should be placed after paragraph 2 to continue the discussion about early bathing.

Response: We have placed the paragraph on early bathing just after paragraph 2

28. In the skin-to-skin care paragraph (#6) I suggest you discuss the reasons found in other studies to explain the lack of skin to-skin care.

Response: We have discussed the reasons of lack of skin to skin care based results from other studies.

29. Limitations should include that the results cannot be generalized to the population.

Response: We have added the statement “Qualitative findings cannot be generalized to all women in the study area” into limitation so the study

30. I suggest you include a paragraph with recommendations to improve the neonatal thermal care practices in the region studied. Especially for delaying the early first bath and incorporating skin-to skin care.

Response: We have added a paragraph dealing with recommendations to improve thermal care practice in study area.

31 Conclusion: There is no mention of your findings regarding immediate breastfeeding.

Response: we have included findings about immediate breastfeeding initiation into our conclusion
