# Supplementary material for: Assessment of neonatal thermal cares: Practices and beliefs among rural women in West Guji Zone, South Ethiopia: A cross-sectional study
Source: PLOS Glob Public Health. 2022 Jun 15;2(6):e0000568. doi: 10.1371/journal.pgph.0000568 (PMC10021890; doi:10.1371/journal.pgph.0000568)
Supplement: Attachment — Submitted filename: Response to Reviewers.docx [file pgph.0000568.s005.docx]

**Response to editors and Reviewers**

**Editor:**

**Journal Requirements:**

Please review your reference list to ensure that it is complete and correct. If you have cited papers that have been retracted, please include the rationale for doing so in the manuscript text, or remove these references and replace them with relevant current references. Any changes to the reference list should be mentioned in the rebuttal letter that accompanies your revised manuscript. If you need to cite a retracted article, indicate the article’s retracted status in theReferences list and also include a citation and full reference for the retraction notice.

**Response**: We have reviewed our references and they are complete and correct. Not retrcated reference has been cited.

**1. Reviewer #2:** This is a much stronger paper, congratulations. However, the Results section needs some minor changes to better convey the findings. My suggestion continues to be to combine both qualitative and quantitative results. You have done some of that but it is not consistent throughout the “Women’s Practice of Neonatal Thermal Care” subsection. Most of the integration is reduced to mentioning what was found in the focus groups leaving all evidence in the next section. This makes the Results section repetitive and short on evidence sometimes. I apologize if my previous recommendation around this wasn’t clear. I think the paper will benefit from reorganizing the Results section in the following way:

1.    Socio-demographic Characteristics of Respondents

2.     Past Obstetrics History of Studied Women

3.    **Early bath**:

**4.**    **Drying and wrapping:**

**5.**    **Head covering and Immediate breastfeeding:**

6.   **Skin to skin care:**.

**Response**: Thank you very for the suggestion to re-organize the results section. We found your suggestion very useful to convey the findings in more logical way. So we re-organized the results section as follows:

1. **Socio-demographic Characteristics of Respondents**: contain the same information as the previous submission.
2. **Past Obstetrics History of Studied Women**: contain the same information as the previous submission.
3. **Early bathing, drying and wrapping of newborns**: We merge this two together because the information are closely and FGDs participants mostly presented their beliefs regarding bathing, drying and wrapping of newborns together. so the following is what we present under this subtopic:

------------------------------------------------------------------------------------------------------------

**Early bathing, Drying and Wrapping of Newborns**

Majority, 350(90.2%) of women had prepared clothes for immediate drying of babies before delivery. However, only 32(8.2%) of newborns were dried before delivery of placenta. Significant proportion of women, 131(33.8%) and 135(35.3%) performed drying of their babies immediately after placental delivery and a long time after placental delivery respectively. A qualitative finding shows that drying is usually done after the first newborn bath. Focus group discussants said like these “*immediately after delivery before drying, newborns are bathed and then wrapped with clean clothes. But if a delivery is at a health facility, newborns are not bathed. They are wrapped with clothes and no bath is done until mothers return their home from the health facility*”- 18 years old and mother of 5 months old infant… “*Some women perform drying and wrapping. But this practice is at its early state of expansion among women who have exposure to health facilities. The common practice is to bath the baby immediately after delivery with warm water and, then wrap with warm clothes*” -18 years old and mother of 2 months old infant. It is also believed that early newborn bath before drying and wrapping, followed by wrapping with warm and clean clothes prevents cold. Focus group discussants quoted “*early bathing is better than drying and wrapping in preventing cold”*-20 years old and mother of 5 months old infant….“*Early bathing and then wrapping with clean clothes prevents cold*”-18 years old and mother of 5 months old infant. FGD participants also mentioned cold prevention as the reason for performing drying and wrapping.

The main reason for performing the first bath before wrapping and drying is to remove birth fluids from newborn as these fluids have bad odor and hated by people. It is also believed that if not removed, birth secretions expose newborn to cold. Therefore to avoid bad odor and cold, bathing is done before wrapping and drying. For instance, FGD participants mentioned two main reasons of early newborn bath, which are directly or indirectly related to cold prevention. The first reason is to remove malodorous birth secretions, so that drying and wrapping can be done to prevent cold. Similar to this, 82.3% of survey participants mentioned that they bathed newborns early to remove different secretions or blood or dirty secretion from body. The second reason is the belief that, if not removed, birth secretions attract cold. Therefore women perform early bath to prevent cold.

Great majority, 328(84.5 %) of women have bathed their baby in less than 6 hours of delivery and 306(78.9%) women used warm water for the first bath. Forty two (70.0%) of women who delayed the first newborn bath at least by 6 hours after delivery did so because they were advised by health workers to do so, whereas only 8(11.7%) of them delayed the first bath because they believe it keeps newborns warm. Table 3 presents newborns’ first bath time among rural women in west Guji Zone.

**Table 3. Newborns’ first bath time among rural women in west Guji Zone., Oromia; southern Ethiopia, April 2019**

| The first newborn bath practices | Frequency | Percentage (%) | |
| --- | --- | --- | --- |
| The first bath time after delivery (n=388) |  |  | |
| In less than 6 hours | 328 | 84.5 | |
| after 6 hours | 60 | 15.5 | |
| Type of water used for the first bath(=388) |  |  | |
| Warm water | 306 | 78.9 | |
| Cold water | 61 | 15.7 | |
| others | 21 | 5.4 | |
| Reason for delayed newborn bath(n=60) |  |  | |
| To keep baby warm | 8 | 13.3 | |
| Health professional advised me to do so | 42 | 70.0 | |
| Others | 10 | 16.7 | |
| Reason for early newborn bathing (n=328)* |  |  | |
| To remove different secretions or blood or dirty from body | 270 | 82.3 | |
| To remove visible vernix | 62 | 18.9 | |
| To improve health and strength of newborn | 18 | 5.5 | |
| To make baby refreshed and comfortable | 26 | | 7.9 |
| Encouraging sleep and improving health | 15 | | 4.6 |
| To prevent different smells | 33 | | 10.1 |
| Others | 4 | | 1.2 |

*- the percentage may not add up to 100 as responses are not mutual exclusive

The first newborn bath is usually done after placental delivery. The focus group discussants said like these “*newborns are not bathed before placental delivery*”- 25 years old and mother of 3 months old infant…“*newborns are bathed after placental delivery. Before placental delivery nobody baths baby*”- 19 years old and mother of 5 months old infant. These findings are in congruent with quantitative finding which reported waiting placental delivery, attendants focusing on mothers until placental delivery and waiting newborn get bathed as reasons for delayed wrapping and drying (Table4). When placental delivery is delayed, newborns are wrapped in clothes until placental delivery before the first bath. Here is quote from focus group discussant “*nobody pays attention to baby’s bath before placental delivery. Babies are simply wrapped with clothes without having bath until placental delivery*”-29 years old and mother of 3 months old infant. Qualitative findings also indicated that newborns are immediately dried and wrapped without having the first bath if delivery took place in a health facility. This might be due to advises of health workers.

Early bathing of newborns as soon as possible after placental delivery is deep rooted practice. Women strongly believe that newborns should be bathed as soon as possible after placental delivery. Focus discussants quoted “*if placenta is safely delivered, the newborns should be bathed immediately and kept away from cold*”-25 years old and mother of 5 months old infant… “*If placenta is immediately delivered, then the baby will be bathed immediately. Then there will be no problem of cold*”- 22 years old and mother of 5 months old infant.

Majority, 336(86.6%) of women had prepared clothes for immediate wrapping of their baby before delivery. However, only 113(8.2%) of newborns were wrapped within 5 minutes of delivery. Table 4 presents women’s practice of immediate drying and wrapping of newborns.

**Table 4. Newborn’s immediate drying and wrapping practices among rural women in West Guji Zone, Oromia; southern Ethiopia, April 2019**

| Newborn drying and wrapping practices | Frequency | Percentage (%) | |
| --- | --- | --- | --- |
| First drying time (n=388) |  |  | |
| Immediately before placental delivery | 32 | 8.2 | |
| Immediately after placental delivery | 131 | 33.7 | |
| Long time after placental delivery | 137 | 35.3 | |
| Cannot remember | 88 | 22.7 | |
| First drying time in minute(n=388) |  |  | |
| In less than 5 minutes | 92 | 23.7 | |
| Between 5-15 minutes | 125 | 32.2 | |
| Between 16-30 minutes | 51 | 13.1 | |
| After 30 minutes | 57 | 14.7 | |
| Could not remember | 63 | 16.2 | |
| Reason for delayed drying (n=233) |  |  | |
| Attendant was focusing on mother until placenta delivery | 80 | 34.3 | |
| Waited until umbilical cord is cut | 17 | 7.3 | |
| Waited until placental delivery | 74 | 31.8 | |
| Drying baby is taboo until placental burial | 27 | | 11.6 |
| Nobody was available | 21 | | 9.0 |
| Waited until baby get bath | 14 | | 6.00 |
| First wrapping time in minute(n=388) |  | |  |
| In less than 5 minutes | 113 | | 29.1 |
| Between 5-15 minutes | 68 | | 17.5 |
| Between 16-30 minutes | 38 | | 9.8 |
| After 30 minutes | 117 | | 30.1 |
| Could not remember | 52 | | 13.4 |
| Reason for delayed wrapping(n=223) |  | |  |
| Attendant was focusing on mother until placenta delivery | 45 | | 20.2 |
| Waited until umbilical cord is cut | 28 | | 12.5 |
| Waited until placental delivery | 43 | | 19.3 |
| Wrapping baby is taboo until placental burial | 6 | | 2.7 |
| Nobody was available | 15 | | 6.7 |
| Waited until baby get bath | 86 | | 38.6 |

Regarding immediate drying of newborn which is usually done after the first bath, women believe that drying with dry clothes damages fragile skin of newborns. Instead of immediate drying, earlier bathing is done to remove birth secretions. A focus group discussant quoted “*because newborn’s skin is very fragile, it should not be touched by clothes for drying. That is why bath is done to remove birth secretions*”-36 years old and mother of 4 months old infant. Immediate drying before bathing is more commonly done by women who have exposure to health facility and it is at its early stage of expansion.

-------------------------------------------------------------------------------------------------------------------.

4. **Head covering and immediate breastfeeding of Newborns:** The following is what we present under this subtopic:

**-**---------------------------------------------------------------------------------------------…

**Head Covering and Immediate Breastfeeding of Newborns**

Two hundred sixty eight(69.1%) women put head covering to their baby immediately after delivery and almost all of them, 267(68.8%) maintained the practice during neonatal period. Qualitative assessment shows that head covering is done to prevent cold as well as sun exposure. Two hundred sixty six (68.6%) women had prepared head covering when reached term. All women believe in importance of head covering to prevent cold from newborns. Also immediate covering of head of newborns as soon as possible is believed to be important practice. Focus group discussants quoted “*Yes it is helpful. It prevents cold from injuring baby*”-33 years old and mother of 5 months old infant… “*Head covering should be done immediately after delivery*”- 28 years old and mother of 5 months old infant.

Three hundred sixty four (93.8%) women breastfed their baby and 210(57.7%) of them initiated breast feeding within one hour of delivery. Exclusive breast feeding during neonatal period was practiced by 227(62.4%) women. The FGD participants did not mention any thermal protection benefits associated with breastfeeding including early initiation and exclusive breastfeeding. The reasons for early initiation of breastfeeding that FGD participants mentioned are that because they see it from their seniors and the fear that newborns may not adapt to breastfeeding if breastfeeding is delayed. Table 5 presents rural women’s breast feeding practice after delivery in west Guji zone.

**Table 5. Rural women’s practice of breast feeding during neonatal period, West Guji Zone, Oromia; southern Ethiopia, April 2019**

| breast feeding practices | Frequency | Percentage (%) |
| --- | --- | --- |
| Time to initiate breast feeding after delivery (n=364) |  |  |
| Within one hour | 210 | 57.7 |
| after one hour | 154 | 42.3 |
| What did you do with colostrum?(=364) |  |  |
| Provided to baby | 136 | 37.4 |
| Discarded | 226 | 62.1 |
| others | 2 | 0.6 |
| Did you provide extra foods/fluids during neonatal period (n=388) |  |  |
| Yes | 137 | 35.3 |
| No | 227 | 58.5 |
| Could not remember | 24 | 6.2 |
| Reasons for delayed initiation of breast feeding within 1 hour of delivery (n=154) |  |  |
| Colostrum is dirty/not good for baby | 28 | 18.2 |
| Lack of sufficient breast milk | 10 | 6.5 |
| Baby need sleep/rest after delivery | 55 | 35.7 |
| Baby did not show sign of hunger | 10 | 6.5 |
| Baby has to be bathed | 13 | 8.4 |
| Mother has to rest | 18 | 11.7 |
| Mother needed bathing | 10 | 6.5 |
| Others | 10 | 6.5 |

Though initiation of breastfeeding is almost universal and women claim that they initiate breast feeding immediately after placental delivery and after the first bath, they did not mention or believed in thermal care importance of either early or frequent breast feeding. Focus group discussants quoted “*I do not think that early initiation of breast feeding helps in keeping baby warm except that delayed initiation of breastfeeding results in baby’s failure to adapt with breastfeeding*”-20 years old and mother of 5 months old infant… “*Early initiation of breastfeeding does not prevent cold from newborns. Delaying up to 2to 3 hours after delivery without initiating breastfeeding does not have any problem. Just we learned early initiation of breastfeeding from our mothers*”-25 years old and mother of 5 months old infant.

-------------------------------------------------------------------------------------------------------…

5. **Skin to Skin Care of Newborns: The following is what we presented here**

**----------------------------------------------------------------------------------------------------------------…**

**Skin to Skin Care of Newborns**

Skin to skin care of newborn is non-existent thermal care practice and is strange to women in the study area. Women perceive skin to skin care of newborn as an odd, frightening and potentially dangerous ways of handling newborns. Focus group discussants quoted “*such practice (*i.e skin to skin care*) is not done in this area. Holding baby in such way is frightening. This practice is new to us. We hear/see it today”-*20 years old and mother of 5 months old infant… “*If skin to skin care is done, babies may get injured especially by inexperienced young mothers*”- 30 years old and mother of 4 months old infant… “*Because we are strange to it (*i.e skin to skin care*), it looks somewhat odd to us*”- 20 years old and mother of 3 months old infant. Elder women would not advise younger women to do skin to skin care. Furthermore, women do not believe that skin to skin care helps in keeping newborn warm. However they claimed that they can do skin to skin care of newborns if they are taught to do so.

--------------------------------------------------------------------------------------------------------------------

**2. Reviewer #2**: Finally, on page 13 you said “women believes”. I think you meant “women believe”

**Response**: Yes, We have corrected it. Thank you so much.
