# Supplementary material for: Assessment of neonatal thermal cares: Practices and beliefs among rural women in West Guji Zone, South Ethiopia: A cross-sectional study
Source: PLOS Glob Public Health. 2022 Jun 15;2(6):e0000568. doi: 10.1371/journal.pgph.0000568 (PMC10021890; doi:10.1371/journal.pgph.0000568)
Supplement: Attachment — Submitted filename: Response to Reviewers.docx [file pgph.0000568.s006.docx]

**Response to editors and Reviewers**

**Editor:**

**Journal Requirements:**

Please review your reference list to ensure that it is complete and correct. If you have cited papers that have been retracted, please include the rationale for doing so in the manuscript text, or remove these references and replace them with relevant current references. Any changes to the reference list should be mentioned in the rebuttal letter that accompanies your revised manuscript. If you need to cite a retracted article, indicate the article’s retracted status in theReferences list and also include a citation and full reference for the retraction notice.

**Response**: We have reviewed our references and they are complete and correct. Not retrcated reference has been cited.

**Editor:** Please provide an Author Summary. This should appear in your manuscript between the Abstract (if applicable) and the Introduction, and should be 150–200 words long. The aim should be to make your findings accessible to a wide audience that includes both scientists and non-scientists.

**Response**: Author summary is included into the manuscript.

**1. Reviewer #2:** You did a decent job with two out of three new sections. However, the main subsection, “Early bathing, drying and wrapping of newborns”, needs further work. There is little articulation between paragraphs, some topics are repetitive (e.g., early bath as a measure to prevent cold), grammar could significantly improve, direct quotation is not based on Vancouver’s citation style, some paragraphs have a different format (double space vs. 1.5 space), and some findings are not supported with data. Here is an idea of some of the changes you could implement to improve the aforementioned subsection based on my understanding of your current version::

**Response**: Thank you so much for your continued critical input. We undertake the following revisions to the subsections “Early bathing, Drying and Wrapping of Newborns” to convey the findings in more logical and non-repetitive way. So we re-organized the results section as follows:

- We removed all unnecessary repetition within the subsections
- We carefully proofread all the whole document including this subsection and corrected all language errors found
- We made all paragraphs 1.5 spaced.

Thus this sub-section appears as follows:

---------------------------------------------------------------------------------------------------------------------

**Early bathing, Drying and Wrapping of Newborns**

Despite majority of women preparing clothes for drying (90.2%) and wrapping(86.6%) of newborns before delivery, only 32(8.2%) of newborns were dried before delivery of the placenta, and 133(29.1%) of newborns were wrapped within 5 minutes of delivery. A significant proportion of women, 266(68.5%) performed drying of their babies after placental delivery respectively. A qualitative finding shows that drying and wrapping are usually done after the first newborn bath in community settings. However, if delivery is conducted in a health facility, or if placental delivery is delayed, newborns are immediately dried and wrapped without having the first bath. An 18 years old mother of five months old infant said “*immediately after delivery before drying, newborns are bathed and then wrapped with clean clothes. But if delivery is at a health facility, newborns are not bathed. They are wrapped with clothes and no bath is done until mothers return their home from the health facility*”. Here are other quotes from focus group discussants “*Some women perform drying and wrapping. But this practice is at its early state of expansion among women who have exposure to health facilities. The common practice is to bathe the baby immediately after delivery with warm water, and then wrap it with warm clothes.*” (18 years old and mother of 2 months old infant)...“*nobody pays attention to baby’s bath before placental delivery. Babies are simply wrapped with clothes without having a bath until placental delivery.*”(29 years old and mother of 3 months old infant). These findings are in congruent with quantitative findings, which reported waiting for newborns to get bathed as one of the reasons for delayed drying and wrapping (Table 3).

Although FGD participants mentioned cold prevention as the reason for performing drying and wrapping, they believe that drying with dry clothes damages the delicate skin of newborns. Instead of immediate drying, immediate bathing is done to remove birth secretions. A focus group discussant said “*because newborn’s skin is very fragile, it should not be touched with clothes for drying. That is why bath is done to remove birth secretions.*”(36 years old and mother of 4 months old infant).

****New Table 3****

The great majority, 328(84.5 %) of women have bathed their baby in less than 6 hours of delivery, and 306(78.9%) of women used warm water for the first bath. Forty two (70.0%) of women who delayed the first newborn bath at least by 6 hours after delivery did so because they were advised by health workers to do so, whereas only 8(11.7%) of them delayed the first bath because they believe it keeps newborns warm. Table 4 presents newborns’ first bath time among rural women in west Guji Zone.

****New Table 4****

The first newborn bath is usually done after placental delivery. The focus group discussants said “*newborns are not bathed before placental delivery.*”(25 years old and mother of a 3 months old infant)…“*newborns are bathed after placental delivery. Before placental delivery nobody bathes baby.*” (19 years old and mother of a 5 months old infant).

It is believed that an early newborn bath before drying and wrapping followed by wrapping with warm and clean clothes prevents cold. Focus group discussants shared “*early bathing is better than drying and wrapping in preventing cold.” (*20 years old and mother of 5 months old infant)…“*Early bathing and then wrapping with clean clothes prevents cold.*”(18 years old and mother of 5 months old infant).

The main reason for performing early bath of newborns is to remove birth fluids from newborns as these fluids have a bad odor and hated by people. It is also believed that if not removed, birth secretions expose newborns to cold. “*Women hate the smell of birth fluids and say that these fluids attract cold. That is why immediate bathing with warm water is common”,* said 22 years old and mother of 6 months infant. Similarly, 18 years old and mother of 3 months infant shared “*I am not convinced with the concept of delay bathing. For example, do you feel good if a baby stays without getting cleaned from birth’s blood for a day*?” Therefore, to avoid bad odor and cold, immediate bathing is done. FGD participants mentioned two main reasons for immediate newborn bath, which are directly or indirectly related to cold prevention. The first reason is to remove malodorous birth secretions so that drying and wrapping can be done to prevent cold. Similar to this, 82.3% of survey participants mentioned that they bathed newborns early to remove different secretions or blood or dirty secretion from the body. The second reason is the belief that if not removed, birth secretions attract cold. Therefore women perform early bath to prevent cold.

Early bathing of newborns as soon as possible after placental delivery is deep-rooted practice. Women strongly believe that newborns should get a bath as soon as possible after placental delivery. Focus discussants said “*if placenta is safely delivered, the newborns should be bathed immediately and kept away from cold.*”(25 years old and mother of 5 months old infant)… “*If placenta is immediately delivered, then the baby will be bathed immediately. Then, there will be no problem of cold.*”(22 years old and mother of 5 months old infant).

---------------------------------------------------------------------------------------------------------------------

**Reviewer #2**: …….DO YOU HAVE ANY QUOTES ABOUT PREVENTING BAD ODOR?

**Response**: Yes, we have added quotes related to bad odor to appropriate part with the sub-section.
